# Dynamics in Circulating Proinflammatory Biomarkers for Prognostic Assessment of Patients With Advanced HCC – A Substudy From the SORAMIC Trial

**DOI:** 10.3389/fgstr.2022.939192

**Published:** 2022-07-05

**Authors:** Kerstin Schütte, Juozas Kupčinskas, Egidijus Morkunas, Osman Öcal, Regina Schinner, Max Seidensticker, Enrico N. De Toni, Najib Ben Khaled, Maciej Pech, Daniel Palmer, Thomas Berg, Christian Sengel, Bristi Basu, Juan W. Valle, Julia Benckert, Antonio Gasbarrini, Bruno Sangro, Peter Malfertheiner, Jens Ricke

**Affiliations:** ^1^ Department of Gastroenterology, Hepatology and Infectious Diseases, Otto-von-Guericke University, Magdeburg, Germany; ^2^ Department of Internal Medicine and Gastroenterology, Niels-Stensen-Kliniken Marienhospital, Osnabrück, Germany; ^3^ Department of Gastroenterology Hepatology and Endocrinology, Hannover Medical School, Hannover, Germany; ^4^ Institute for Digestive Research and Department of Gastroenterology, Medical Academy, Lithuanian University of Health Sciences, Kaunas, Lithuania; ^5^ Department of Radiology, University Hospital, Ludwig-Maximilians-University (LMU) Munich, Munich, Germany; ^6^ Department of Medicine II, University Hospital, LMU Munich, Munich, Germany; ^7^ Department of Radiology and Nuclear Medicine, Otto-von-Guericke University, Magdeburg, Germany; ^8^ Molecular and Clinical Cancer Medicine, University Hospitals & Clatterbridge, University of Liverpool, Liverpool, United Kingdom; ^9^ Division of Hepatology, Department of Medicine II, Leipzig University Medical Center, Leipzig, Germany; ^10^ Radiology Department, Grenoble University Hospital, La Tronche, France; ^11^ Department of Oncology, University of Cambridge, Cambridge, United Kingdom; ^12^ Division of Cancer Sciences and Department of Medical Oncology, The Christie National Health Service (NHS) Foundation Trust, University of Manchester, Manchester, United Kingdom; ^13^ Charité – Universitätsmedizin Berlin, corporate member of Freie Universität Berlin and Humboldt-Universität zu Berlin, Department of Hepatology and Gastroenterology, Berlin, Germany; ^14^ Fondazione Policlinico Universitario Gemelli IRCCS, Universita Cattolica del Sacro Cuore, Roma, Italy; ^15^ Liver Unit, Clinica Universidad de Navarra and Centro de Investigación Biomédica en Red de Enfermedades Hepáticas y Digestivas (CIBEREHD), Pamplona, Spain

**Keywords:** IL-8, IL-6, biomarker, prognosis, HCC, LPS, VEGF

## Abstract

**Introduction:**

Prediction of response to treatment in patients with advanced hepatocellular carcinoma (HCC) may assist in the selection of personalized management.

**Objective:**

This exploratory analysis of the palliative arm of the SORAMIC trial (ClinicalTrials.gov NCT01126645) evaluated the prognostic potential of basal and dynamic changes in systemic levels of interleukin 6 (IL-6), interleukin 8 (IL-8), systemic vascular endothelial growth factor (VEGF), and lipopolysaccharide (LPS).

**Methods:**

We evaluated the correlations between overall survival (OS) and concentrations of IL-6, IL-8, VEGF, and LPS at follow-up approximately 7-9 weeks after treatment initialization (FU) compared to baseline (BL) in 90 patients treated either with ^90^Yttrium (^90^Y) microspheres combined with sorafenib (n = 44) or with sorafenib (n = 46) alone.

**Results:**

Changes in IL-6 concentration during treatment showed correlations with the outcome. An increase in IL-6 concentration of less than 16.8 pg/mL over baseline readings was associated with better survival [median OS 16.3 months compared with 8.9 months (p = 0.0354)]. Correlations with survival were not observed for VEGF or LPS concentrations at baseline, at FU, or changes between these time points.

**Conclusions:**

Changes in IL 6 serum levels at 7-9 weeks after treatment initialization but not in IL 8, VEGF, or LPS add important information on the outcome of advanced HCC patients treated palliatively within the SORAMIC trial.

## Introduction

The predominant causes of hepatocellular carcinoma (HCC) in Europe are alcoholic liver disease, non-alcoholic steatohepatitis (NASH), and chronic Hepatitis B or C virus infection (1). Development of HCC is regarded as closely linked to a state of chronic inflammation, independent of its cause, and end-stage inflammatory liver disease is commonly associated with liver cirrhosis. Intrahepatic and systemic inflammatory events interact in the development of HCC. The tumor microenvironment (TME) with its local pro-inflammatory and pro-fibrotic elements promotes hepatic carcinogenesis. Several circulating inflammatory cytokines, including interleukin-1α (IL-1α), IL-1β, IL-6, IL-8, and tumor necrosis factor-α (TNF-α), participate in chronic hepatic inflammation and contribute to the neoplastic transformation of hepatocytes (2, 3).

Within the prospective randomized multicenter SORAMIC clinical study patients with advanced HCC were randomized to receive treatment with sorafenib with or without ^90^Yttrium (^90^Y) radioembolization. In previous substudies of the SORAMIC trial we confirmed that baseline IL-6 and IL-8 levels could correlate with survival outcomes of sorafenib-treated patients with HCC (4, 5). In addition, baseline IL-6 showed predictive value for overall survival in patients with advanced HCC undergoing radioembolization (4, 5). This exploratory analysis evaluated whether dynamic changes in systemic levels of the cytokines IL-6 and IL-8 could add prognostic value over basal concentrations. The relevance of VEGF and LPS levels was also assessed in this cohort.

## Methods

The cohort evaluated in this exploratory sub-analysis comprised patients within the palliative treatment arm of the randomized, controlled, multicenter phase II SORAMIC study, which evaluated the impact of ^90^Y selective internal radiation therapy (SIRT) combined with sorafenib compared to sorafenib alone on survival in patients with advanced HCC (6).

**Figure 1:**
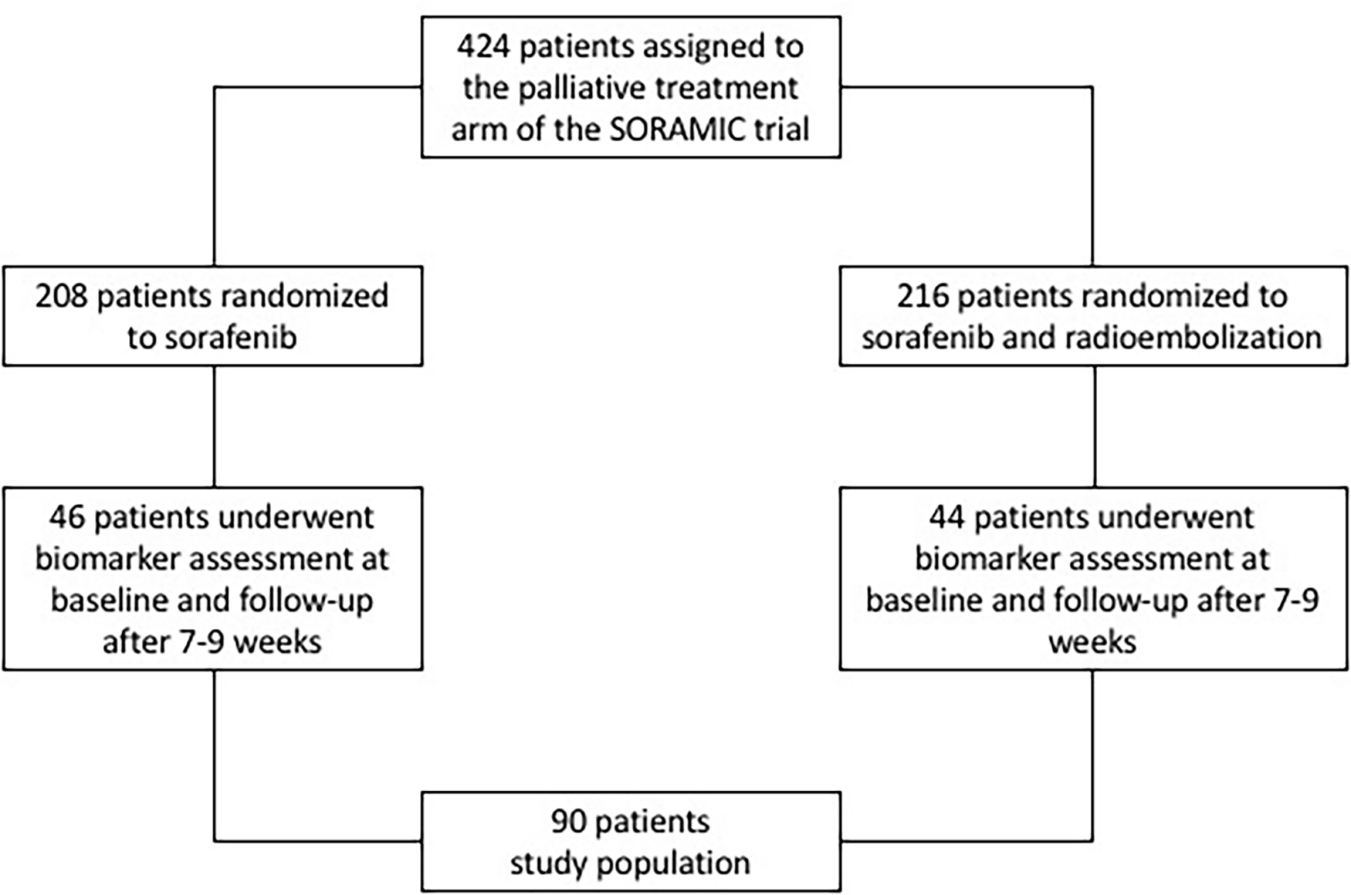
Consort diagram of the study population.

Patients were included in this analysis if they received study treatment within the palliative arm of SORAMIC and took part in the translational program of the study with blood sample analysis at baseline and at first follow-up at approximately 7-9 weeks of treatment (FU). Of the 424 patients randomized after assignment to the palliative arm, 90 fulfilled these criteria within the intention to treat (ITT) population, comprising 46 patients treated with sorafenib alone and 44 patients treated with the combination of SIRT and sorafenib (**Figure 1**). No statistically significant differences were present between the two treatment cohorts with respect to age, gender distribution, presence of liver cirrhosis, liver function, or tumor stage according to Barcelona Clinic Liver Cancer (BCLC) stage (patient characteristics in **Table 1**).

**Table 1:** Baseline characteristics of cohort.

	Total (N=90)	SIRT/Sorafenib (N=44)	Sorafenib (N=46)	P-value
**Gender**				0.7396
Female	7 (7.8)	3 (6.8)	4 (8.7)	
Male	83 (92.2)	41 (93.2)	42 (91.3)	
**Age**				0.2743
Mean (SD)	65.1 (8.3)	64.1 (8.6)	66.1 (8.1)	
Median (IQR)	64.5 (14.0)	63.5 (13.5)	66.0 (13.0)	
**HCC Etiology/Liver Cirrhosis**				
** Alcohol etiology**	47 (52.2)	24 (54.5)	23 (50.0)	0.6661
** Hepatitis B etiology**	4 (4.4)	0	4 (8.7)	0.0454
** Hepatitis C etiology**	19 (21.1)	10 (22.7)	9 (19.6)	0.7133
** Liver Cirrhosis**	76 (84.4)	36 (81.8)	40 (87.0)	0.5014
**Child Pugh**				0.4489
5	56 (62.2)	30 (68.2)	26 (56.5)	
6	25 (27.8)	11 (25.0)	14 (30.4)	
7-8	9 (10.0)	3 (6.8)	6 (13.0)	
**BCLC**				0.7149
BCLC A/B	23 (25.6)	12 (27.3)	11 (23.9)	
BCLC C	67 (74.4)	32 (72.7)	35 (76.1)	
**Number of Lesions**				0.3671
1	6 (6.7)	2 (4.5)	4 (8.7)	
2	9 (10.0)	3 (6.8)	6 (13.0)	
3-30	17 (18.9)	11 (25.0)	6 (13.0)	
diffuse	58 (64.4)	28 (63.6)	30 (65.2)	
**Max. Diameter of Largest Lesion**				0.0518
Nmiss (%)	3 (3.3)	2 (4.5)	1 (2.2)	
Mean (SD)	66.3 (40.7)	75.2 (46.1)	58.0 (33.4)	
Median (IQR)	55.0 (53.0)	69.0 (59.0)	51.0 (29.0)	
**Further disease classification**				
Liver Dominant Disease	86 (95.6)	41 (93.2)	45 (97.8)	0.2852
Extrahepatic Metastases	16 (17.8)	11 (25.0)	5 (10.9)	0.0797
Portal Vein Invasion	46 (51.1)	19 (43.2)	27 (58.7)	0.1411
**Bilirubin at Baseline [µmol/L]**				0.3396
Mean (SD)	15.0 (8.2)	14.2 (9.3)	15.8 (6.9)	
Median (IQR)	13.6 (10.3)	11.2 (11.1)	15.2 (9.5)	
**Albumin at Baseline [g/L]**				0.0514
Mean (SD)	38.8 (6.5)	40.1 (4.6)	37.5 (7.7)	
Median (IQR)	39.1 (6.8)	40.0 (7.4)	37.7 (7.7)	
**Albi Value at Baseline**				0.0202
Mean (SD)	-2.6 (0.6)	-2.7 (0.5)	-2.4 (0.7)	
Median (IQR)	-2.5 (0.7)	-2.8 (0.7)	-2.4 (0.7)	
**Albi Score at Baseline**				0.1222
Grade 1 (Median survival 18.5-85.6 months)	42 (46.7)	25 (56.8)	17 (37.0)	
Grade 2 (Median survival 5.3-46.5 months)	47 (52.2)	19 (43.2)	28 (60.9)	
**Sorafenib: Days Treated**				0.4034
Mean (SD)	338.8 (289.0)	312.8 (224.2)	363.7 (340.4)	
Median (IQR)	279.0 (299.0)	293.0 (280.5)	266.0 (316.0)	
Min-Max	10.0-1534.0	10.0-1050.0	56.0-1534.0	
**Sorafenib: Daily Dose**				0.0576
Mean (SD)	552.1 (204.6)	510.3 (202.0)	592.1 (201.2)	
Median (IQR)	561.1 (344.6)	464.5 (358.4)	706.1 (314.6)	
**SIRT: Total Activity**				
Mean (SD)		1.8 (0.5)		
Median (IQR)		1.9 (0.5)		
**SIRT: Lobes Treated**				
bilobar treatment		29 (65.9)		
lobar treatment		14 (31.8)		
unspecified		1 (2.3)		

AFP, alpha-fetoprotein; BCLC, Barcelona Clinic Liver Cancer; BMI, body mass index; ECOG, Eastern Cooperative Oncology Group; SIRT, selective internal radiation treatment: Three patients had discontinued sorafenib intake before the follow-up visit (finished 1 day, 35 days, and 50 days before FU).

Serum levels of VEGF, IL-6, IL-8, and LPS were measured with enzyme-linked immunosorbent assay (ELISA) from patients’ serum which were obtained at BL and FU. Human VEGF Quantikine ELISA Kit (DVE00; R&D systems, Minneapolis, MN, USA), Human IL-6 Quantikine ELISA KIT (D6050; R&D systems, MN, USA), Human IL-8/CXCL8 Quantikine ELISA Kit (D8000C, R&D systems, MN, USA), and Human Lipopolysaccharides (LPS) ELISA Kit (CSB-E09945h; Cusabio, China) were used in the study. Optical density evaluations were performed at 450 nm and 570 nm (as the reference) using Tecan Sunrise absorbance microplate reader, concentrations were calculated using four parameter logistic regression (4-PL) curve fitting model.

### Statistical Analyses

All statistical analyses were performed using SAS (SAS version 9.4 for Windows; Copyright SAS Institute Inc., Cary, NC, USA). Numerical data are presented as means with standard deviations. For categorical data, results are given as absolute numbers with percentages. For comparison of categorical data, chi-square tests were applied. T-tests or Mann-Whitney U tests were used for testing homogeneity of independent samples in continuous data. We used receiver operating characteristic (ROC) curves to determine the cut-off value for differences in concentrations of IL-6, IL-8, and VEGF that could produce the highest sensitivity and specificity to predict individual survival shorter than the median overall survival. Univariate and multivariate Cox regression analyses were performed to identify risk factors for mortality. The Kaplan-Meier method was used for estimates of overall survival, and the log-rank test was used to compare survival groups. All tests were carried out two-sided. The level of significance was set to 0.05 without adjusting for multiplicity.

## Results

At data closure for this substudy 72 patients (80%) had died. The median overall survival (OS) of the cohort analyzed within this sub-analysis was 14.5 months: OS was 14.3 months in the sorafenib only arm and 15.0 months in the combined treatment arm (p=0.5964).

IL-6 concentrations at BL (rho = -0.3024, p = 0.0038) and FU (rho = -0.3192, p = 0.0022) negatively correlated with OS.

**Figure 2:**
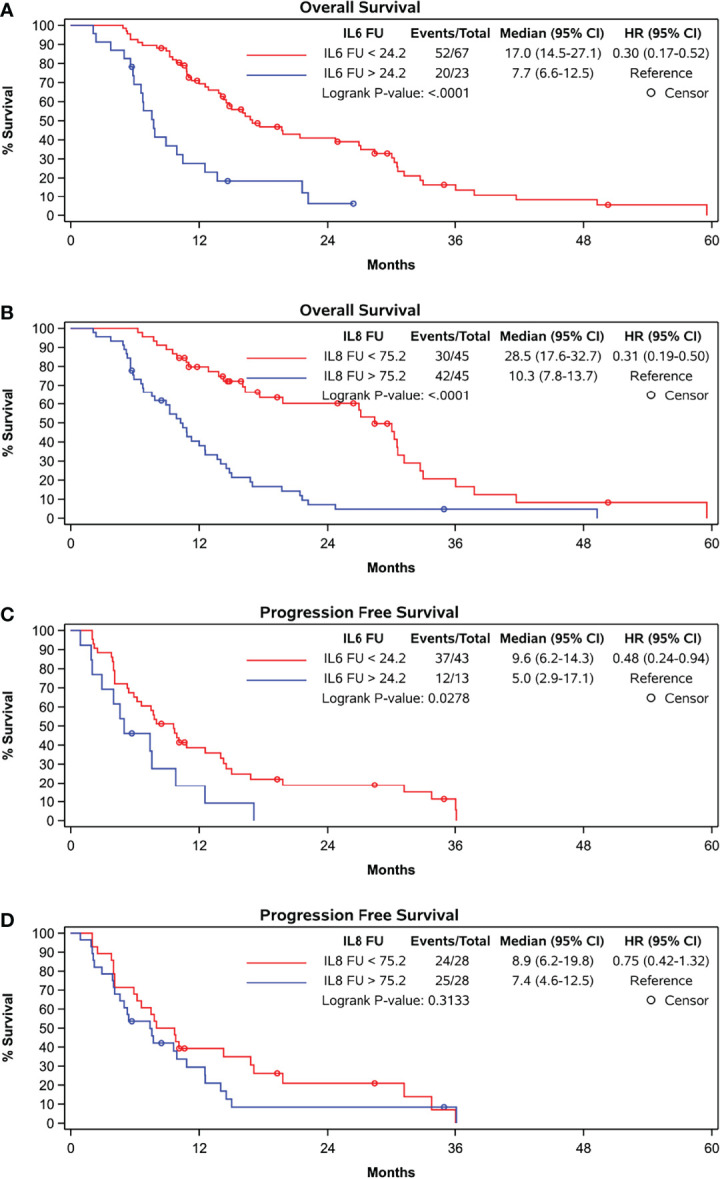
Kaplan-Meier curves grouped by follow-up levels according to cut-off in ROC-curve-analyses **(A)** OS and IL-6, cut-off at 24.2 pg/ml, **(B)** OS and IL-8, cut-off at 75.2 pg/ml, **(C)** PFS and IL-6, cut-off at 24.2 pg/ml, and **(D)** PFS and IL-8, cut-off at 75.2 pg/ml.

ROC curve analysis identified a FU concentration of 24.18 pg/mL best distinguished patients with a median survival longer than 12 months (sensitivity 42.9%, specificity 87.5%). Median OS was 17.0 months if FU IL-6 was lower than 24.18 pg/mL and was 7.7 months in the 23 patients (28% of patients) with higher FU IL-6 levels (p < 0.0001) (**Table 2** and **Figure 2A**). Patients with lower IL-6 at FU also had a significantly longer progression-free survival (median PFS: 9.6 months vs. 5.0 months; p=0.0278) (**Figure 2C**).

**Table 2:** Statistical analyses of prognostic value for overall survival.

Parameter		Descriptive statistics(N=90)				Correlation of IL-6/IL-8/VEGF/LPS with overall survival		Best cut-off from ROC-analysis (Youden Index), distinguishing between OS </>12 months				Kaplan Meier for OS in patients with high vs. low parameter values		
		Mean	SD	Min.	Max.	Spearman Correlation Coefficients	P-Value H0: Rho=0	Cut-Off	Sensitivity	Specificity	Youden Index	Median OS lower than ROC cut-off	Median OS higher than ROC cut-off	P-Value LogRank
IL-6 [pg/ml]	BL	14.88	25.16	1.39	205.71	-0.3024	0.0038	9.70	59.5%	70.8%	0.304	21.41	10.26	<0.0001
	FU	26.95	43.61	2.25	298.60	-0.3192	0.0022	24.18	42.9%	87.5%	0.304	16.95	7.74	<0.0001
	abs. diff.	12.07	31.45	-60.95	138.37	-0.1774	0.0943	16.83	38.1%	85.4%	0.235	16.30	8.89	0.0354
	pct. diff.	183.14	303.49	-76.99	1754.95	-0.0633	0.5536	154.52	45.2%	70.8%	0.161	15.02	10.85	0.2170
IL-8 [pg/ml]	BL	150.96	384.75	2.91	3414.00	-0.3913	0.0001	80.35	64.3%	79.2%	0.435	21.41	9.51	<0.0001
	FU	205.76	409.82	0.15	3328.55	-0.4370	<0.0001	75.25	70.7%	63.6%	0.344	28.46	10.26	<0.0001
	abs. diff.	48.82	538.50	-3358.83	3251.94	-0.0991	0.3667	-29.45	31.7%	95.5%	0.272	10.82	15.02	0.6516
	pct. diff.	179.11	538.59	-99.52	4244.91	-0.0202	0.8544	-23.60	31.7%	81.8%	0.135	26.92	14.52	0.1295
VEGF [pg/ml]	BL	673.86	683.54	5.45	3806.36	-0.1319	0.2180	857.04	39.0%	77.1%	0.161	16.75	12.00	0.2120
	FU	409.12	276.52	73.46	1178.69	-0.1991	0.0600	263.02	71.4%	52.1%	0.235	19.80	12.52	0.0935
	abs. diff.	-261.71	640.29	-3475.61	645.03	0.0115	0.9146	-456.70	31.7%	81.3%	0.130	12.52	16.30	0.5028
	pct. diff.	65.02	283.12	-95.00	1924.31	-0.0059	0.9562	42.31	34.1%	77.1%	0.112	14.82	12.85	0.8798
LPS [pg/ml]	BL	175.00	141.06	7.40	896.08	0.0341	0.7495	58.76	88.1%	22.9%	0.110	17.64	14.26	0.2054
	FU	148.82	110.76	3.00	841.00	-0.1045	0.3298	273.78	100.0%	8.5%	0.085	14.26	18.84	0.6043
	abs. diff.	-27.77	148.91	-666.94	703.65	-0.0991	0.3556	100.30	95.2%	14.9%	0.101	13.80	16.07	0.7488
	pct. diff.	17.74	112.56	-86.73	512.27	-0.1278	0.2326	156.76	100.0%	12.8%	0.128	13.80	31.21	0.0874

IL-6 interleukin 6; IL-8 interleukin-8; VEGF systemic vascular endothelial growth factor; LPS lipopolysaccharide; ROC receiver operating curve; BL baseline; FU follow-up at 7-9 weeks; abs. diff. absolute difference; pct. diff: percentage difference.

**Figure 3:**
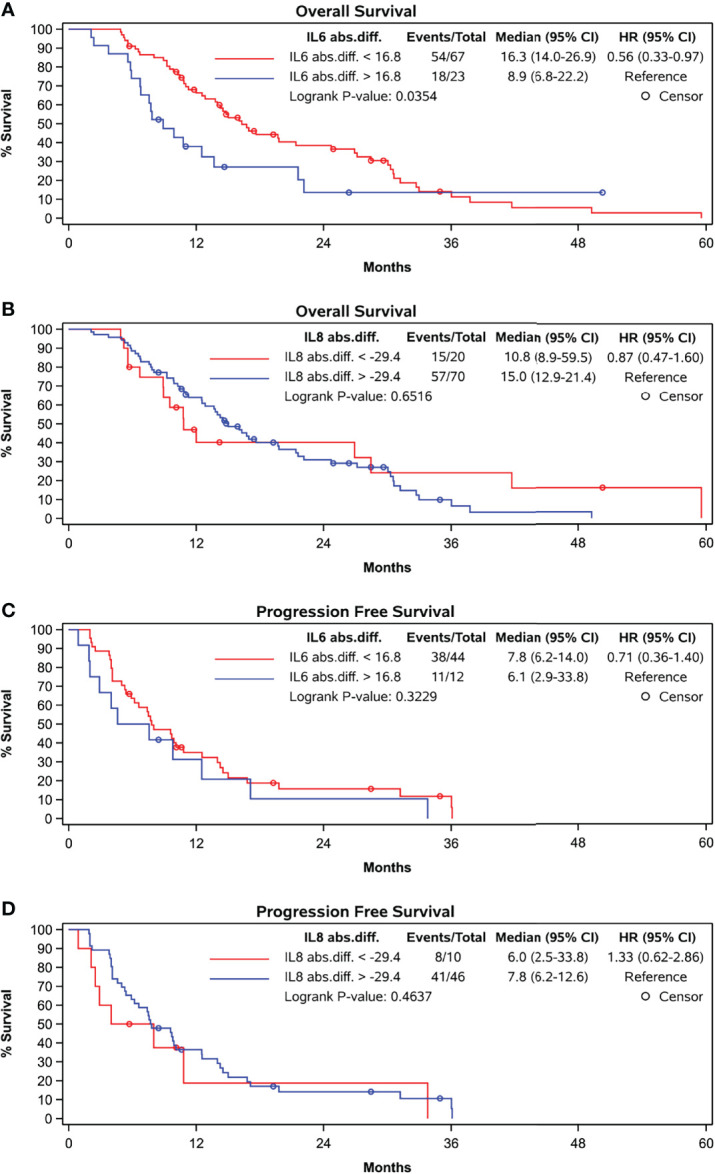
Kaplan-Meier curves grouped by absolute difference between baseline and follow-up levels according to cut-off in ROC-curve-analyses. **(A)** OS and abs. difference in IL-6, cut-off at 16.8 pg/ml, **(B)** OS and abs. difference in IL-8, cut-off at -29.4 pg/ml, **(C)** PFS and abs. difference in IL-6, cut-off at 16.8 pg/ml and **(D)** PFS and abs. difference in IL-8, cut-off at -29.4 pg/ml.

ROC curve analysis identified that absolute increases in IL-6 from BL to FU with a cut-off of 16.8 pg/mL optimally distinguished patients with OS shorter than 12 months from those with an OS exceeding 12 months (sensitivity 38.1%, specificity 85.4%). Patients showing absolute increase in IL-6 concentration less than 16.8 pg/mL had a median OS of 16.3 months versus 8.9 months for those with greater IL-6 increase (p = 0.0354) (**Figure 3A**).

A Cox regression with BL IL-6 as well as absolute IL-6 change from BL to FU in the model showed significant p-values for both effects (BL: p<0.0001; abs. change BL to FU: p=0.0260).

**Figure 4:**
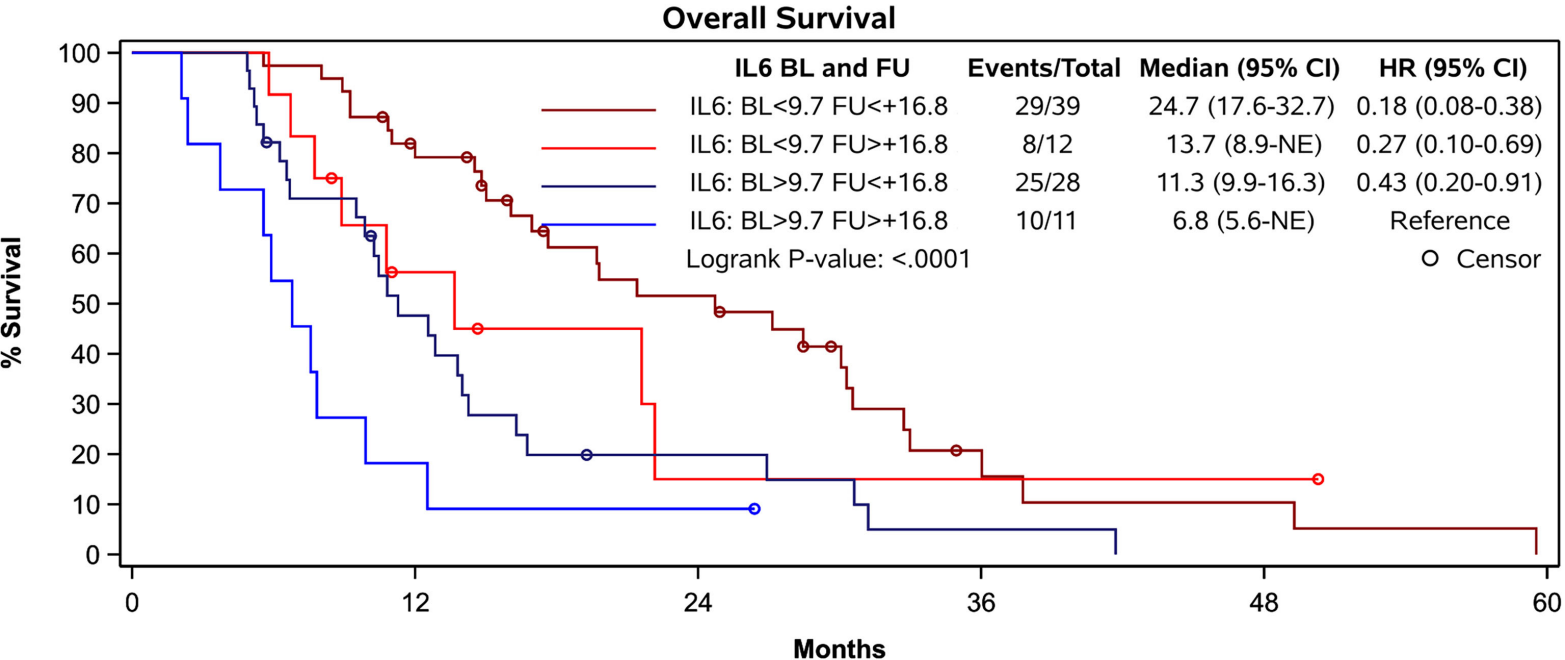
Survival of patients according to their prognostic groups identified by IL-6 levels. Patients with a baseline IL-6 concentration exceeding 9.7 pg/ml and an increase of 16.8 pg/mL or more at FU presented with a median OS of 6.8 months while patients with less pronounced increase showed a median OS of 11.3 months (p = 0.0337). Patients with low IL-6 levels at baseline and increase of less than 16.8 pg/ml at FU had the most favorable prognosis.

In patients with a BL IL-6 concentration < 9.7 pg/mL the additional FU IL-6 did not add prognostic value. However, in patients with a BL IL-6 concentration exceeding 9.7 pg/ml (n=39), the additional FU value identified a subgroup with worse expectation of survival. An increase of more than 16.8 pg/mL at FU was associated with a median OS of only 6.8 months compared with median OS of 11.3 months in those showing a less pronounced increase within this subgroup (p = 0.0337) (**Figure 4**).

The percentage increase in IL-6 concentration did not correlate with OS nor did the absolute or percentage increase in IL-6 concentration correlate with PFS (**Figure 3C**).

Both baseline (rho = -0.3913, p = 0.0001) and FU concentrations (rho = -0.4370, p = <.0001) of IL-8 significantly correlated with OS (**Table 2** zand **Figure 2B**). At follow-up, patients with IL-8 concentrations below the threshold of 75.25 pg/ml had a significantly longer OS than those above threshold (28.5 months compared to 10.3 months, p < 0.0001). However, there was no correlation with PFS at this cut-off (**Figure 2D**).

Furthermore, the absolute or percentage difference in IL-8 concentrations between both time points did not correlate with OS (rho = -0.0991, p = 0.3667) (**Figure 3B**) nor with PFS (**Figure 3D**).

Multivariate Cox regression analyses integrating also treatment modality, liver function, portal vein thrombosis, and tumor distribution revealed that baseline concentrations of IL-6 and IL-8 as well as liver function and the presence of portal vein thrombosis are independent factors impacting on OS, while absolute differences in IL-6 and IL-8 concentrations are not (**Tables 3A, B** and **Supplementary Table S1**).

**Table 3A:** Impact follow-up assessment of IL-6 concentration: univariate and multivariate analysis.

		Parameter estimate	Standard error	P-value	Hazard ratio	95%	Confidence limits for HR
**Univariate** **analyses**	IL6 FU < 24,2	-1.21	0.28	<0.0001	0.30	0.17	0.52
	IL6 abs. diff. < 16,8	-0.58	0.28	0.038	0.56	0.33	0.97
**Multivariate** **analysis**	IL6 BL < 9,7	-0.99	0.27	0.0002	0.37	0.22	0.63
	IL6 abs. diff. < 16,8	-0.53	0.30	0.0747	0.59	0.33	1.05
	SIRT/Sorafenib treatment (vs. Sorafenib only)	0.19	0.25	0.4395	1.21	0.74	1.98
	Child-Pugh A (vs. B)	-2.05	0.44	<0.0001	0.13	0.06	0.31
	Portal Vein Infiltration YES (vs. NO)	-0.64	0.28	0.0207	0.53	0.31	0.91
	Liver-Dominant Disease YES (vs. NO)	-0.72	0.53	0.1754	0.48	0.17	1.38

**Table 3B:** Impact of follow-up assessment of IL-8 concentration: univariate and multivariate analysis.

		Parameter estimate	Standard error	P-value	Hazard ratio	95%	Confidence limits for HR
**Univariate** **analyses**	IL8 FU < 75,2	-1.18	0.25	<0.0001	0.31	0.19	0.50
	IL8 abs. diff. < -29,4	-0.14	0.31	0.6549	0.87	0.47	1.60
**Multivariate** **analysis**	IL8 BL < 80,3	-1.12	0.29	0.0001	0.33	0.19	0.58
	IL8 abs. diff. < -29,4	-0.64	0.35	0.0641	0.53	0.27	1.04
	SIRT/Sorafenib treatment (vs. Sorafenib only)	-0.17	0.26	0.529	0.85	0.50	1.42
	Child-Pugh A (vs. B)	-2.05	0.44	<0.0001	0.13	0.05	0.30
	Portal Vein Infiltration YES (vs. NO)	-0.57	0.28	0.0417	0.57	0.33	0.98
	Liver-Dominant Disease YES (vs. NO)	-0.30	0.55	0.5874	0.74	0.25	2.18

Serum VEGF and LPS concentrations did not show any correlation with survival in this cohort of patients, either at baseline, follow-up level, or the changes between these time points (**Table 2**).

**Figure 5:**
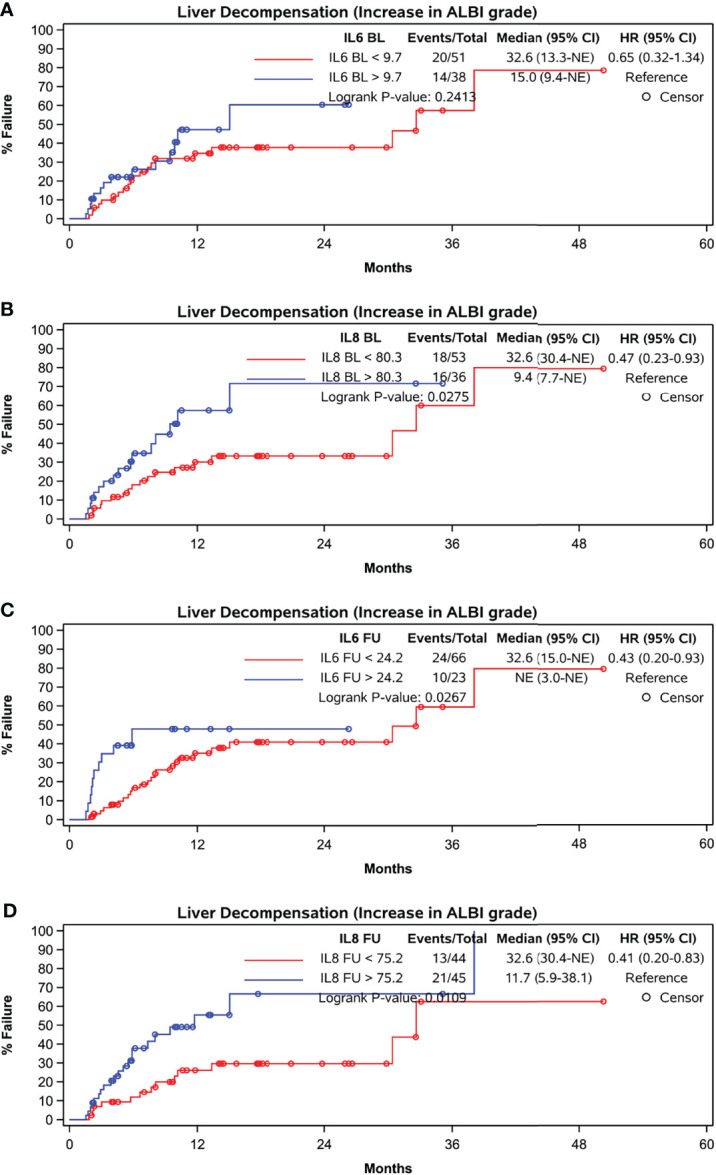
Time to liver function deterioration assessed by ALBI-grade. **(A)** baseline IL-6, cut-off at 9.7 pg/ml, **(B)** baseline IL-8, cut-off at 80.3 pg/ml, **(C)** FU IL-6, cut-off at 24.2 pg/ml, **(D)** FU IL-8, cut-off at 75.2 pg/ml.

While baseline IL-6 levels did not correlate with liver decompensation indicated by an increase in ALBI grade at FU (p = 0.2413), baseline IL-8 concentration (p = 0.0275) as well as IL-6 and IL-8 concentration at FU did (p = 0.0267 and p = 0.0109) (**Figures 5A–D**).

## Discussion

We have previously reported on the prognostic value of baseline IL-6 concentrations in patients treated within the palliative arm of the SORAMIC study (4, 5). This current analysis suggests that repeated measurement of IL-6 7-9 weeks after treatment initialization is of prognostic value in these patients and that dynamic changes in IL-6 concentrations further helps to identify patients with dismal prognosis. An increase of IL-6 levels above 16.8 pg/ml from baseline is associated with poor prognosis. The subgroup of patients with baseline IL-6 readings of 9.7 pg/ml or over and an increase of IL-6 level of more than 16.8 ppg/ml have particularly poor outcomes with median OS of only 6.8 months. Favorable prognosis (median OS of 24.7 months) was associated with low IL-6 levels at baseline and increases of less than 16.8 pg/ml at follow-up. Contrary to initial expectation, no additional value in the prognostic assessment of these palliative HCC patients was conferred by the analysis of the dynamics of IL-8, VEGF, or LPS.

Clinical factors influencing survival of HCC patients in numerous studies including the SORAMIC trial, relate to tumor burden (number and size of HCC nodules within the liver, invasion of portal vein, existence of extrahepatic metastases), clinical performance status, and liver function (1, 6). As previously published, our analysis confirms liver function and portal vein infiltration as prognostic factors in patients with advanced HCC included in the SORAMIC trial (7). A pooled analysis of the two phase III studies that led to approval of sorafenib for treatment of patients with advanced HCC revealed that presence of macrovascular invasion, high alpha-fetoprotein (AFP) levels, and high neutrophil-to-lymphocyte ratio (NLR) were prognostic factors of poorer OS. Patients had a benefit of the treatment with sorafenib irrespective of prognostic factors, although lack of extrahepatic metastases, lower NLR, and chronic hepatitis C virus infection were predictive for greater survival benefit (8, 9). Since the approval of sorafenib, the evaluation of additional systemic biomarkers applicable either in a prognostic or in a predictive manner has been the focus of many studies.

The chronically inflamed microenvironment in the liver induces macrophages to adopt the M2 phenotype and transform into tumor associated macrophages (TAMs) which are responsible for continuous local proinflammatory signaling leading to parenchymal cell transformation (10). TAMs secrete IL-8 which activates the PI3K/AKT/HIF-1a signaling pathway involved in invasiveness and metastatic progress of HCC (10–12). They also secrete further proinflammatory cytokines including IL-6 with antiapoptotic activity in HCC cell lines (12). TAMs stimulate tumor growth also by suppression of the adaptive immune system through expression of high levels of programmed cell death-ligand 1 (PD-L1), and by this mechanism suppress the antitumor cytotoxic T-cell responses (13). Tumor cells also secrete a variety of inflammatory factors and chemokines to recruit TAMs including IL-6, IL-8, and IL-34 (14). Increased levels of proinflammatory cytokines in patients with chronic liver disease are associated with increased risk of HCC and correlate with survival in patients with advanced HCC (15, 16). IL-6 activates signal transducer and activator of transcription 3 (STAT3) which is implicated in induction of sorafenib resistance in patients with HCC (2, 17), but IL-6 levels have also been shown to be a predictor of survival even in patients with liver cirrhosis without HCC (18).

IL-8 in addition to its pro-tumorigenic activity is a potent angiogenic factor and produced by most HCC cell lines (19, 20). The inhibition of IL-8 signaling increases the sensitivity of liver cancer cells to sorafenib (21). We and others have previously reported that baseline IL-8 values predict outcome in patients with advanced HCC treated with sorafenib (5). However, we did not find an add-on value by follow-up measurement or the assessment of dynamics of IL-8 concentration with respect to survival.

The analysis of larger panels focusing on factors relevant to the proinflammatory tumor microenvironment and giving a more holistic view on systemic consequences of chronic tumor associated inflammation might result in more accurate results with respect to individual prognosis in patients with advanced HCC. In recent years, a clinical breakthrough in systemic therapy of HCC was reached with the introduction of checkpoint-inhibitors targeting programmed death-1, programmed death-ligand 1, and cytotoxic T lymphocyte antigen-4. These molecules resolve T-cell activation to maintain inflammatory homeostasis, protect tissue integrity, and prevent unwanted autoimmunity under physiological condition (22). In patients with tumors, the administration of checkpoint inhibitors unleashes tumor-directed cytotoxic T-cells specific against an unknown spectrum of tumor-associated antigens (22, 23). The immune contexture of HCC before treatment is the most promising predictive marker for response to immunotherapies (24). The identification of a subset of patients with high grade of tumor associated inflammation might therefore guide treatment decisions toward early systemic immune-checkpoint inhibition.

Concentrations of IL-6 and IL-8 at FU both were of prognostic value with respect to future deterioration of liver function assessed by the ALBI-Grade (25). This is of special clinical interest, as preserved liver function is the key factor in decisions on applicability of sequential treatments, and the repeated measurement of IL-6 and IL-8 potentially help to identify patients at risk to become untreatable because of impairment of liver function rather than because of tumor progression. In a previous analysis on patients treated within SORAMIC in palliative intent with the combination of radioembolization and sorafenib high IL-6 concentrations at baseline were associated with a significant shorter time to liver dysfunction. Although there was a tendency for a shorter time-to-liver dysfunction in patients with high IL-8 concentrations in that analysis, the result was not significant (4). As in that paper, liver function deterioration was defined by a significant increase in bilirubin levels, cholestasis, and secretory function of the liver are assumed to be the dominant factors in this setting. The current analysis also takes liver synthesis mirrored by albumin concentration into account which might explain differences in the results.

Vascular endothelial growth factor (VEGF), a protein that promotes angiogenesis, supports tumor cell survival, proliferation, and vessel formation has been shown to have prognostic value in HCC patients (26, 27). Sorafenib, a multi-tyrosine kinase inhibitor with inhibitory activity against vascular endothelial growth factor receptor (VEGFR) 2 (28) was the standard treatment in patients with advanced HCC for more than a decade. Baseline concentrations of IL-6 and IL-8 have been demonstrated to be predictive for response to sorafenib monotherapy in patients with advanced HCC (5). Recently, the combination of atezolizumab, a humanized anti-PDL1 antibody to restore antitumor T-cell activity, with bevacizumab, binding to VEGF, has been defined as the new first line treatment standard (29, 30).

Previously, it has been reported that circulating concentrations of VEGF are of prognostic value in patients with HCC undergoing liver resection, liver transplantation, or locoregional therapy (26). A decrease in VEGF levels at week 8 of sorafenib treatment was prognostic for better survival in advanced HCC patients (mostly on the background of chronic viral hepatitis) (31). We however did not observe any prognostic potential of systemic VEGF levels in our cohort. It is unclear whether this discrepancy is related to the patient characteristics within this cohort, with low numbers of patients with viral etiology of HCC.

Alterations in gut microbiota composition are associated with hepatic inflammatory diseases and may play a contributory role in hepatic carcinogenesis (32). Dysbiotic gut microbiota composition leads to increased hepatic exposure with gut-derived microbiota-associated molecular patterns (MAMPs) that include lipopolysaccharides (LPS), a cell wall component of Gram-negative bacteria.

Bacterial compounds reach the liver *via* the portal blood flow and are eliminated by Kupffer cells under normal conditions (33). In a mouse model, LPS levels increase during the course of chronic liver disease fostering proinflammatory responses in the periphery and in the liver (32, 34). Plasma LPS concentrations correlate with the degree of liver dysfunction (35, 36).

An increased exposure to LPS contributes to hepatocarcinogenesis *via* activation of Toll-like receptor-4 signaling in mouse models (37). By this, LPS promote hepatic inflammation, fibrosis, proliferation, and the activation of anti-apoptotic signaling (37). In an analysis of fecal samples of patients with HCC and liver cirrhosis LPS‐producing genera were increased compared to patients with liver cirrhosis (38).

The proinflammatory tumor microenvironment in part induced by pathogen-associated molecular patterns (PAMPs) including LPS also promotes tumor progression. High concentrations of infiltrating TAMs are associated with poor prognosis of HCC (12, 39). However, our study does not support the idea of measuring systemic concentrations of LPS for prognostic purposes in patients receiving treatment with sorafenib with or without radioembolization in patients with advanced HCC. It is likely that concentrations of LPS in the portal blood flow and in the liver are of higher significance for progression of HCC than systemic concentrations.

Our analysis is limited by the rather small number of patients that could be included into the analysis compared to the whole study population of SORAMIC.

## Conclusion

As availability of effective systemic treatment options for advanced HCC patients increases, sequential treatments are now a realistic strategy for management. Methods to expeditiously identify patient groups who do well on particular treatments, or select patients with more dismal prognosis, may help to guide optimal management. Although clinical factors have previously been utilized in this aim, the baseline assessment of proinflammatory cytokines IL-6 and IL-8 in addition to inclusion of the dynamic changes of IL-6 values at 7-9 weeks after treatment initiation (sorafenib with or without radioembolization used in the SORAMIC trial) appears of value in the prediction of patients who do better on palliative treatments within the SORAMIC study. Early re-assessment of IL-6 and IL-8 may also help to identify patients at risk for deterioration of liver function which potentially precludes further lines of treatment.

## Data Availability Statement

The raw data supporting the conclusions of this article will be made available by the authors, without undue reservation.

## Ethics Statement

The studies involving human participants were reviewed and approved by Ethical committee Otto-von-Guericke University Magdeburg, Germany and the local ethics committees. The patients/participants provided their written informed consent to participate in this study.

## Author Contributions

KS, PM, and JR: Conception and design of the study; Generation, collection, assembly, analysis, and/or interpretation of data; Drafting or revision of the manuscript; Approval of the final version of the manuscript. JK, EM, OÖ, RS, MS, ED, NBK, MP, DP, TB, CS, BB, JV, JB, AG, and BS: Generation, collection, assembly, analysis, and/or interpretation of data; Drafting or revision of the manuscript; Approval of the final version of the manuscript.

## Funding

SORAMIC is an investigator-initiated trial sponsored by the University of Magdeburg. Financial support was granted by Sirtex Medical and Bayer Healthcare. This research was supported by the NIHR Cambridge Biomedical Research Centre (BRC-1215-20014*).

## Conflict of Interest

KS: Personal fees: Bayer. MS: Grants: Sirtex, Bayer; Personal fees: Sirtex, Bayer. ED: ED has served as a paid consultant for AstraZeneca, Bayer, BMS, EISAI, Eli Lilly & Co, Pfizer, IPSEN, and Roche. He has received reimbursement of meeting attendance fees and travel expenses from Arqule, Astrazeneca, BMS, Bayer, Celsion and Roche, and lecture honoraria from BMS and Falk. He has received third-party funding for scientific research from Arqule, AstraZeneca, BMS, Bayer, Eli Lilly, and Roche. NB: NB has received reimbursement of meeting attendance fees and travel expenses from EISAI and lecture honorarium from Falk. MP: Personal fees: Sirtex, Bayer. TB: Receipt of grants/research supports: Abbvie, BMS, Gilead, MSD/Merck, Humedics, Intercept, Merz, Norgine, Novartis, Orphalan, Sequana Medical. Receipt of honoraria or consultation fees/advisory board: Abbvie, Alexion, Bayer, Gilead, GSK, Eisai, Enyo Pharma, Falk Foundation, HepaRegeniX GmbH, Humedics, Intercept, Ipsen, Janssen, MSD/Merck, Novartis, Orphalan, Roche, Sequana Medical, SIRTEX, SOBI, and Shionogi Participation in a company sponsored speaker's bureau: Abbvie, Alexion, Bayer, Gilead, Eisai, Intercept, Ipsen, Janssen, MedUpdate GmbH, MSD/Merck, Novartis, Orphalan, Sequana Medica, SIRTEX, and SOBI. JV: Dr. Valle reports personal fees from Agios, personal fees from AstraZeneca, personal fees from Baxter, personal fees from Genoscience Pharma, personal fees from Hutchison Medipharma, personal fees from Imaging Equipment Ltd (AAA), personal fees from Incyte, personal fees from Ipsen, personal fees from Mundipharma EDO, personal fees from Mylan, grants, personal fees and non-financial support from NuCana, personal fees from QED, personal fees from Servier, personal fees from Sirtex, personal fees from Zymeworks, outside the submitted work. PM: Grants: Bayer, Sirtex. JR: Sirtex, Bayer; Personal fees: Sirtex, Bayer.

The remaining authors declare that the research was conducted in the absence of any commercial or financial relationships that could be construed as a potential conflict of interest.

## Publisher’s Note

All claims expressed in this article are solely those of the authors and do not necessarily represent those of their affiliated organizations, or those of the publisher, the editors and the reviewers. Any product that may be evaluated in this article, or claim that may be made by its manufacturer, is not guaranteed or endorsed by the publisher.

## Supplementary Material

Supplementary Figure 1 Boxplots indicating concentrations of IL-6, IL-8, VEGF and LPS, each comparing absolute levels at baseline and follow-up stratified by treatment, overall survival and a combination of both.
