# Supplementary figures and images for: Dynamics in Circulating Proinflammatory Biomarkers for Prognostic Assessment of Patients With Advanced HCC – A Substudy From the SORAMIC Trial
Source: Front Gastroenterol (Lausanne). 2022 Jul 5;1:939192. doi: 10.3389/fgstr.2022.939192 (PMC12952395; doi:10.3389/fgstr.2022.939192)

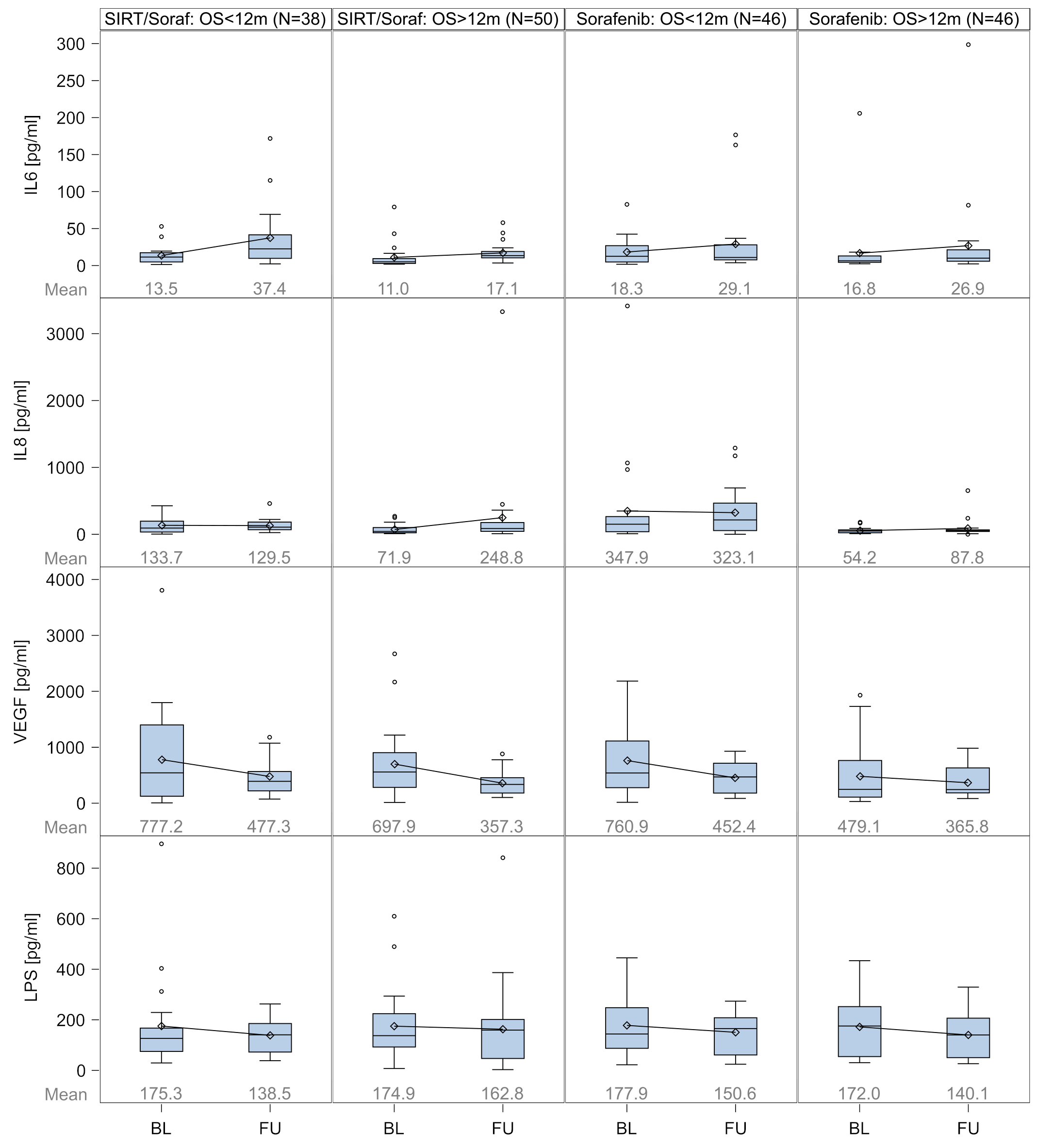

Supplement: Supplementary Figure 1 — Boxplots indicating concentrations of IL-6, IL-8, VEGF and LPS, each comparing absolute levels at baseline and follow-up stratified by treatment, overall survival and a combination of both. [file Image_1.jpeg]
