# Supplementary material for: Dynamics in Circulating Proinflammatory Biomarkers for Prognostic Assessment of Patients With Advanced HCC – A Substudy From the SORAMIC Trial
Source: Front Gastroenterol (Lausanne). 2022 Jul 5;1:939192. doi: 10.3389/fgstr.2022.939192 (PMC12952395; doi:10.3389/fgstr.2022.939192)
Supplement: Supplementary file 2 [file Table_1.docx]

|  | | | Total | SIRT/ Sorafenib | Sorafenib | P-Value | OS  ≤ 12 months | OS  > 12months | P-Value | Child Pugh A | Child Pugh B | P-Value | PVI | no PVI | P-Value | liver dominant | not liver dominant | P-Value |
| --- | --- | --- | --- | --- | --- | --- | --- | --- | --- | --- | --- | --- | --- | --- | --- | --- | --- | --- |
|  |  |  | (N=90) | (N=44) | (N=46) |  | (N=42) | (N=48) |  | (N=80) | (N=10) |  | (N=46) | (N=44) |  | (N=86) | (N=4) |  |
| **IL6** | BL | < 9.7 | 51 (56.7) | 28 (63.6) | 23 (50.0) | 0.1919 | 17 (40.5) | 34 (70.8) | 0.0037 | 49 (61.3) | 2 (20.0) | 0.0131 | 20 (43.5) | 31 (70.5) | 0.0098 | 49 (57.0) | 2 (50.0) | 0.7831 |
|  |  | ≥ 9.7 | 39 (43.3) | 16 (36.4) | 23 (50.0) |  | 25 (59.5) | 14 (29.2) |  | 31 (38.8) | 8 (80.0) |  | 26 (56.5) | 13 (29.5) |  | 37 (43.0) | 2 (50.0) |  |
|  | FU | < 24.2 | 67 (74.4) | 32 (72.7) | 35 (76.1) | 0.7149 | 25 (59.5) | 42 (87.5) | 0.0024 | 63 (78.8) | 4 (40.0) | 0.0081 | 31 (67.4) | 36 (81.8) | 0.1168 | 64 (74.4) | 3 (75.0) | 0.9792 |
|  |  | ≥ 24.2 | 23 (25.6) | 12 (27.3) | 11 (23.9) |  | 17 (40.5) | 6 (12.5) |  | 17 (21.3) | 6 (60.0) |  | 15 (32.6) | 8 (18.2) |  | 22 (25.6) | 1 (25.0) |  |
|  | abs. diff. | < 16.8 | 67 (74.4) | 31 (70.5) | 36 (78.3) | 0.3960 | 26 (61.9) | 41 (85.4) | 0.0107 | 63 (78.8) | 4 (40.0) | 0.0081 | 33 (71.7) | 34 (77.3) | 0.5474 | 63 (73.3) | 4 (100) | 0.2306 |
|  |  | ≥ 16.8 | 23 (25.6) | 13 (29.5) | 10 (21.7) |  | 16 (38.1) | 7 (14.6) |  | 17 (21.3) | 6 (60.0) |  | 13 (28.3) | 10 (22.7) |  | 23 (26.7) |  |  |
|  | pct. diff. | < 154.5 | 57 (63.3) | 23 (52.3) | 34 (73.9) | 0.0332 | 23 (54.8) | 34 (70.8) | 0.1145 | 52 (65.0) | 5 (50.0) | 0.3534 | 31 (67.4) | 26 (59.1) | 0.4140 | 54 (62.8) | 3 (75.0) | 0.6204 |
|  |  | ≥ 154.5 | 33 (36.7) | 21 (47.7) | 12 (26.1) |  | 19 (45.2) | 14 (29.2) |  | 28 (35.0) | 5 (50.0) |  | 15 (32.6) | 18 (40.9) |  | 32 (37.2) | 1 (25.0) |  |
| **IL8** | BL | < 80.3 | 53 (58.9) | 24 (54.5) | 29 (63.0) | 0.4128 | 15 (35.7) | 38 (79.2) | <.0001 | 51 (63.8) | 2 (20.0) | 0.0080 | 20 (43.5) | 33 (75.0) | 0.0024 | 52 (60.5) | 1 (25.0) | 0.1588 |
|  |  | ≥ 80.3 | 37 (41.1) | 20 (45.5) | 17 (37.0) |  | 27 (64.3) | 10 (20.8) |  | 29 (36.3) | 8 (80.0) |  | 26 (56.5) | 11 (25.0) |  | 34 (39.5) | 3 (75.0) |  |
|  | FU | < 75.2 | 45 (50.0) | 18 (40.9) | 27 (58.7) | 0.0916 | 13 (31.0) | 32 (66.7) | 0.0007 | 43 (53.8) | 2 (20.0) | 0.0442 | 21 (45.7) | 24 (54.5) | 0.3990 | 44 (51.2) | 1 (25.0) | 0.3063 |
|  |  | ≥ 75.2 | 45 (50.0) | 26 (59.1) | 19 (41.3) |  | 29 (69.0) | 16 (33.3) |  | 37 (46.3) | 8 (80.0) |  | 25 (54.3) | 20 (45.5) |  | 42 (48.8) | 3 (75.0) |  |
|  | abs. diff. | < -29.4 | 20 (22.2) | 11 (25.0) | 9 (19.6) | 0.5353 | 14 (33.3) | 6 (12.5) | 0.0177 | 17 (21.3) | 3 (30.0) | 0.5303 | 13 (28.3) | 7 (15.9) | 0.1589 | 19 (22.1) | 1 (25.0) | 0.8913 |
|  |  | ≥ -29.4 | 70 (77.8) | 33 (75.0) | 37 (80.4) |  | 28 (66.7) | 42 (87.5) |  | 63 (78.8) | 7 (70.0) |  | 33 (71.7) | 37 (84.1) |  | 67 (77.9) | 3 (75.0) |  |
|  | pct. diff. | < -23.6 | 26 (28.9) | 14 (31.8) | 12 (26.1) | 0.5487 | 14 (33.3) | 12 (25.0) | 0.3842 | 23 (28.8) | 3 (30.0) | 0.9345 | 15 (32.6) | 11 (25.0) | 0.4260 | 25 (29.1) | 1 (25.0) | 0.8606 |
|  |  | ≥ -23.6 | 64 (71.1) | 30 (68.2) | 34 (73.9) |  | 28 (66.7) | 36 (75.0) |  | 57 (71.3) | 7 (70.0) |  | 31 (67.4) | 33 (75.0) |  | 61 (70.9) | 3 (75.0) |  |
| **VEGF** | BL | < 857 | 63 (70.0) | 31 (70.5) | 32 (69.6) | 0.9267 | 26 (61.9) | 37 (77.1) | 0.1170 | 59 (73.8) | 4 (40.0) | 0.0281 | 32 (69.6) | 31 (70.5) | 0.9267 | 61 (70.9) | 2 (50.0) | 0.3719 |
|  |  | ≥ 857 | 27 (30.0) | 13 (29.5) | 14 (30.4) |  | 16 (38.1) | 11 (22.9) |  | 21 (26.3) | 6 (60.0) |  | 14 (30.4) | 13 (29.5) |  | 25 (29.1) | 2 (50.0) |  |
|  | FU | < 263 | 37 (41.1) | 15 (34.1) | 22 (47.8) | 0.1856 | 12 (28.6) | 25 (52.1) | 0.0237 | 36 (45.0) | 1 (10.0) | 0.0339 | 16 (34.8) | 21 (47.7) | 0.2122 | 33 (38.4) | 4 (100) | 0.0143 |
|  |  | ≥ 263 | 53 (58.9) | 29 (65.9) | 24 (52.2) |  | 30 (71.4) | 23 (47.9) |  | 44 (55.0) | 9 (90.0) |  | 30 (65.2) | 23 (52.3) |  | 53 (61.6) |  |  |
|  | abs. diff. | < -456.7 | 22 (24.4) | 10 (22.7) | 12 (26.1) | 0.7108 | 13 (31.0) | 9 (18.8) | 0.1790 | 18 (22.5) | 4 (40.0) | 0.2247 | 11 (23.9) | 11 (25.0) | 0.9045 | 20 (23.3) | 2 (50.0) | 0.2237 |
|  |  | ≥ -456.7 | 68 (75.6) | 34 (77.3) | 34 (73.9) |  | 29 (69.0) | 39 (81.3) |  | 62 (77.5) | 6 (60.0) |  | 35 (76.1) | 33 (75.0) |  | 66 (76.7) | 2 (50.0) |  |
|  | pct. diff. | < 42.3 | 65 (72.2) | 34 (77.3) | 31 (67.4) | 0.2955 | 28 (66.7) | 37 (77.1) | 0.2710 | 57 (71.3) | 8 (80.0) | 0.5603 | 31 (67.4) | 34 (77.3) | 0.2955 | 62 (72.1) | 3 (75.0) | 0.8990 |
|  |  | ≥ 42.3 | 25 (27.8) | 10 (22.7) | 15 (32.6) |  | 14 (33.3) | 11 (22.9) |  | 23 (28.8) | 2 (20.0) |  | 15 (32.6) | 10 (22.7) |  | 24 (27.9) | 1 (25.0) |  |
| **LPS** | BL | < 58.8 | 17 (18.9) | 8 (18.2) | 9 (19.6) | 0.8669 | 6 (14.3) | 11 (22.9) | 0.2967 | 16 (20.0) | 1 (10.0) | 0.4462 | 10 (21.7) | 7 (15.9) | 0.4800 | 16 (18.6) | 1 (25.0) | 0.7494 |
|  |  | ≥ 58.8 | 73 (81.1) | 36 (81.8) | 37 (80.4) |  | 36 (85.7) | 37 (77.1) |  | 64 (80.0) | 9 (90.0) |  | 36 (78.3) | 37 (84.1) |  | 70 (81.4) | 3 (75.0) |  |
|  | FU | < 273.8 | 86 (95.6) | 41 (93.2) | 45 (97.8) | 0.2852 | 42 (100) | 44 (91.7) | 0.0556 | 76 (95.0) | 10 (100) | 0.4695 | 44 (95.7) | 42 (95.5) | 0.9637 | 82 (95.3) | 4 (100) | 0.6590 |
|  |  | ≥ 273.8 | 4 (4.4) | 3 (6.8) | 1 (2.2) |  |  | 4 (5.5) |  | 4 (5.0) |  |  | 2 (4.3) | 2 (4.5) |  | 4 (4.7) |  |  |
|  | abs. diff. | < 100.3 | 81 (90.0) | 40 (90.9) | 41 (89.1) | 0.7786 | 40 (95.2) | 41 (85.4) | 0.1213 | 71 (88.8) | 10 (100) | 0.2636 | 41 (89.1) | 40 (90.9) | 0.7786 | 77 (89.5) | 4 (100) | 0.4952 |
|  |  | ≥ 100.3 | 9 (10.0) | 4 (9.1) | 5 (10.9) |  | 2 (4.8) | 7 (14.6) |  | 9 (11.3) |  |  | 5 (10.9) | 4 (9.1) |  | 9 (10.5) |  |  |
|  | pct. diff. | < 156.8 | 84 (93.3) | 41 (93.2) | 43 (93.5) | 0.9551 | 42 (100) | 42 (87.5) | 0.0177 | 74 (92.5) | 10 (100) | 0.3700 | 43 (93.5) | 41 (93.2) | 0.9551 | 80 (93.0) | 4 (100) | 0.5845 |
|  |  | ≥ 156.8 | 6 (6.7) | 3 (6.8) | 3 (6.5) |  |  | 6 (12.5) |  | 6 (7.5) |  |  | 3 (6.5) | 3 (6.8) |  | 6 (7.0) |  |  |
